# Supplementary material for: Non-resonant power-efficient directional Nd:YAG ceramic laser using a scattering cavity
Source: Nat Commun. 2021 Jan 4;12:8. doi: 10.1038/s41467-020-20114-2 (PMC7782720; doi:10.1038/s41467-020-20114-2)
Supplement: Supplementary file 1 — Supplementary Information [file 41467_2020_20114_MOESM1_ESM.pdf]

Supplementary Information for:

Non-resonant power-efficient directional Nd:YAG ceramic laser using a scattering cavity

Lee and Ma et al.

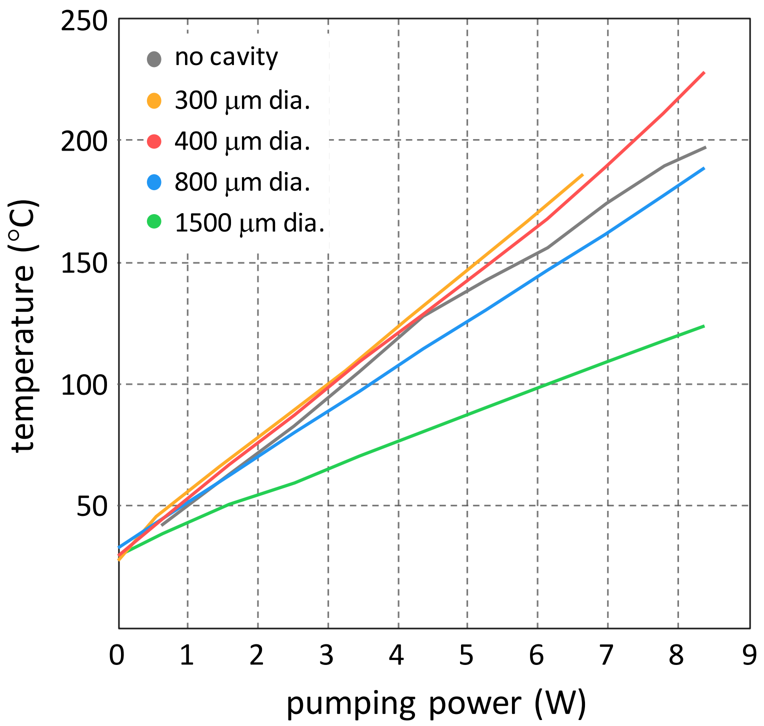

Supplementary Figure 1 | Estimated cavity temperature. The cavity temperature is calculated from the peak wavelengths. The theoretical model proposed in Supplementary Ref. [1] is used.

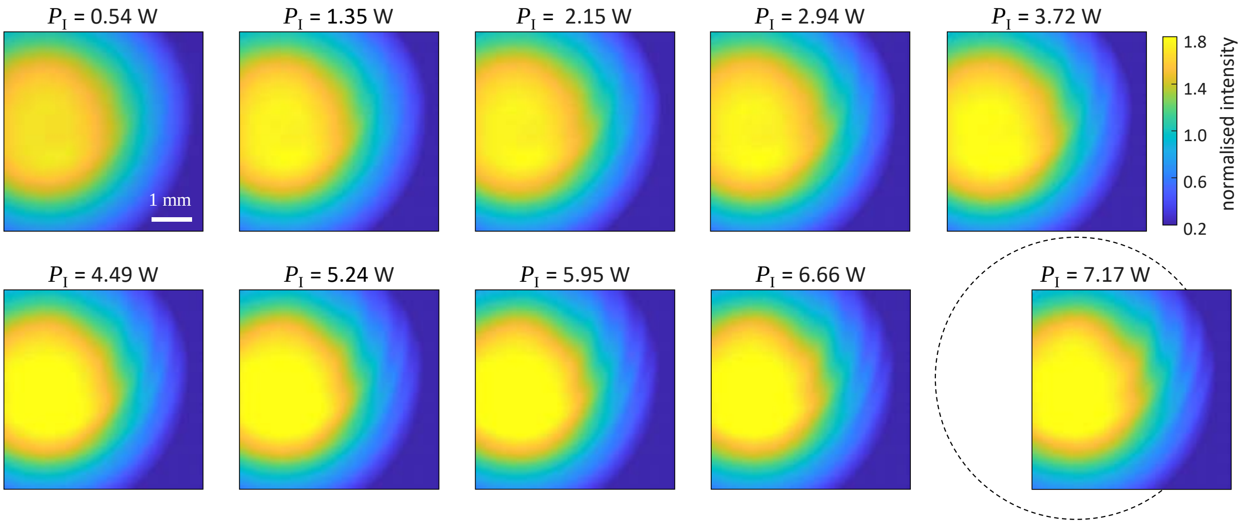

Supplementary Figure 2 | Beam spatial profiles at various pumping powers (P_I). Each measured image was normalised to its mean value. Dotted circle extrapolates the boundary of the beam profile. The decrease in the visibility of Newton's rings is observed for the low pumping power as the laser linewidth broadens. This interference effect is originated from the protection glass of the used image sensor, and is not the intrinsic property of the laser beam. The initial beam profile is confined to the fiber core. After passing the coupling and collimating lenses, the size of the beam is magnified to have a diameter of 10 mm and larger, and subsequently collimated. Because the beam size is larger than a typical image sensor, we placed an additional 4f system with a magnification factor of 0.5 to examine the spatial profile of the laser beam.

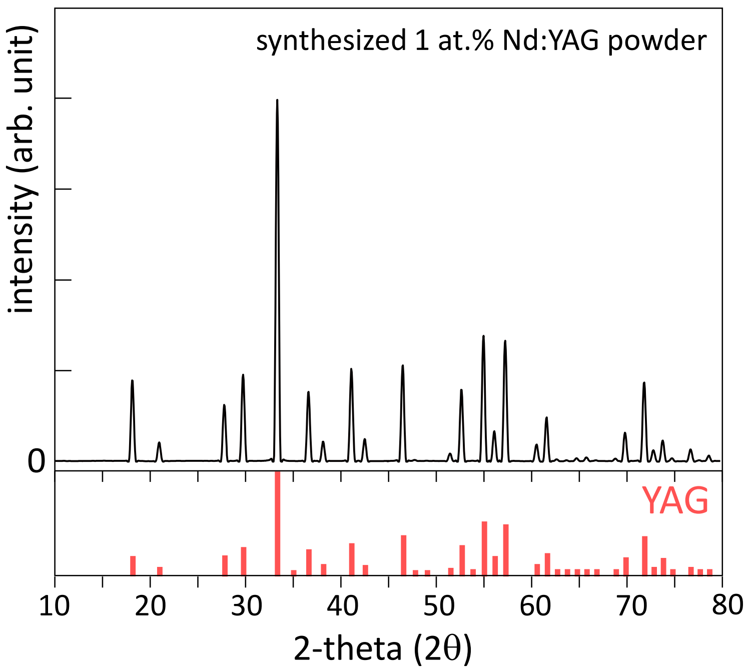

Supplementary Figure 3 | The X-ray diffraction (XRD) result of synethesized powder. The measured XRD result agrees well with the expected cubic YAG phase.

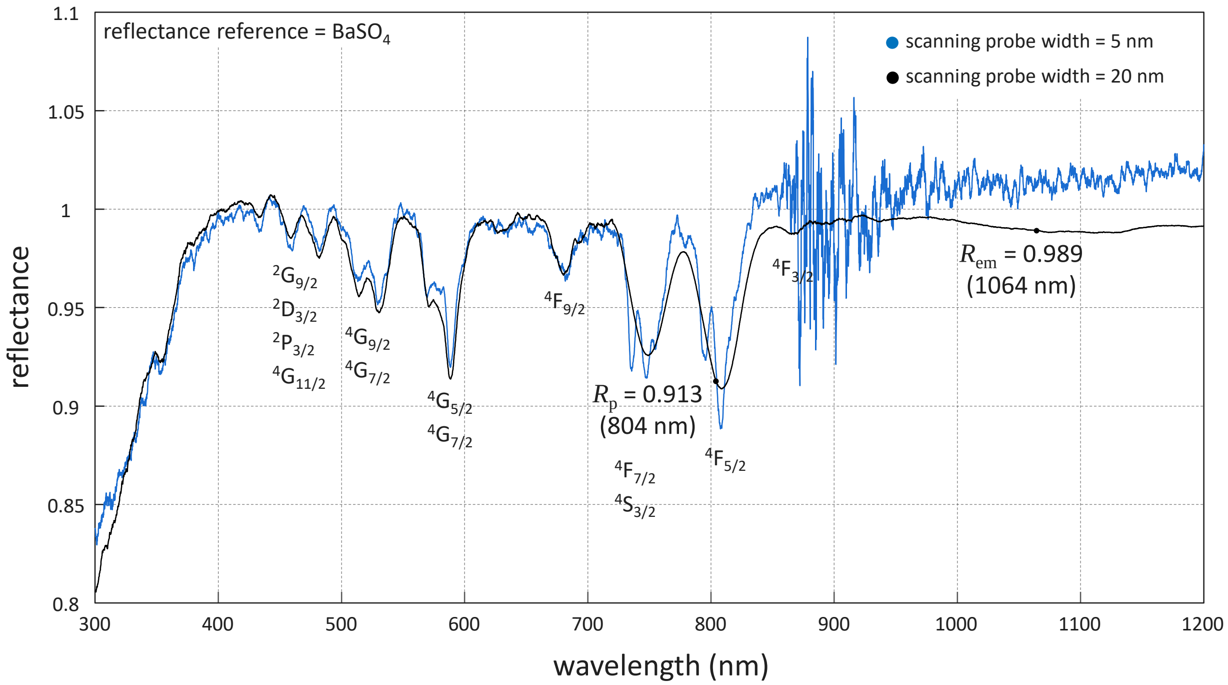

Supplementary Figure 4 | The diffuse reflectance of fabricated porous ceramic. The diffuse reflectance is measured in a wavelength range of 300 to 1200 nm (SolidSpec-3700, Shimadzu). Adjusting the slit size, the linewidth of the scanning probe can be determined. Because the smaller scanning probe width gives rather noisy and biased results, the 20 nm scanning probe width is used for the reflectance measurement, and the 5 nm width is used to see the high-resolution features. $BaSO_4$ plate is used as a reflectance reference. Additional reflectance measurements with coherent Nd:YAG laser (1064.1 nm) is also used as the other reference point for the precise reflectance measurement.

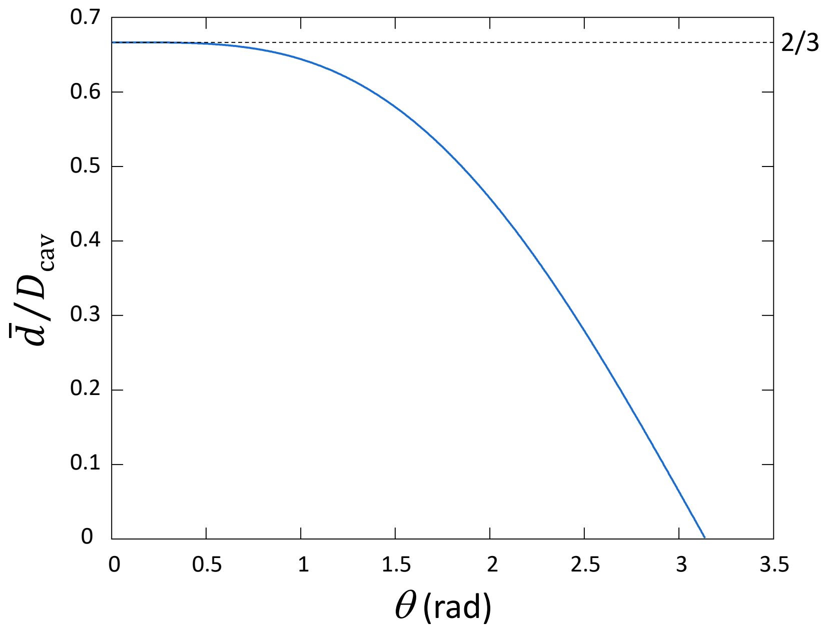

Supplementary Figure 5 | Numerically calculated the mean distance between wall reflections. The mean distance between wall reflections ($\bar{d}$) is numerically calculated using the cavity diameter ($D_{\rm cav}$) as a unit. Notice $\theta = \sin^{-1}\left(D_{\rm a}/D_{\rm cav}\right)$ for $0 < \theta \le \pi/2$ (the cases we are interested in), and $\theta = \pi - \sin^{-1}\left(D_{\rm a}/D_{\rm cav}\right)$ for $\pi/2 < \theta \le \pi$ (see Fig. 6a).

Supplementary Note 1. The derivation and generalized version of Equations (2) **and** (3)

In order to calculate the pumping efficiency (η_{p}) and the output coupling efficiency (η_{oc}), we first calculate the the effective reflectance of the cavity ($R_{\mathrm{eff}}^{\mathrm{p}}$ or $R_{\mathrm{eff}}^{\mathrm{em}}$) that presents the mean probability that the light reflects back to the intracavity space whenever the light meets internal cavity surface. It can be calculated by the sum of (*statistical probability*) × (*reflectance*) = $\sum_k p_k R_k$ of possible reflective events. In this work, we considered the four possible reflective events as shown in Fig. 3b: (i) reflection loss by the wall, (ii) emission outcoupling, (iii) loss through the aperture, (iv) undesired leaks, which leads

$$R_{\mathrm{eff}}^{\mathrm{p,em}} = p_{\mathrm{wall}} R_{\mathrm{wall}}^{\mathrm{p,em}} + p_{\mathrm{oc}} R_{\mathrm{oc}} + p_{\mathrm{noc}} R_{\mathrm{noc}} + p_{\mathrm{leak}} R_{\mathrm{leak}} , \tag{1}$$

where p and R are the statistical probability and reflectance of the events that the light can experience in the cavity; the subscripts 'wall', 'oc', 'noc', and 'leak' correspond to the events (i)-(iv) of Fig. 6b, respectively; and the superscripts 'p' and 'em' denote the used pumping and emission light, respectively. We assumed the reflectances that are independent to the used scattering meidum (R_{oc} and R_{noc}) are identical for pumping and emission lights. The reflectance of undesired leaks are set to zero ($R_{\mathrm{leak}} = 0$) without losing the generality.

Similarly, the total loss chance for a single intracavity reflection event is the sum of (*statistical probability*) × (1 – *reflectance*) = $\sum_k p_k \left(1 - R_k\right)$ = $\sum_k p_k - \sum_k p_k R_k$ $= 1 - R_{\mathrm{eff}}$. Then, the pumping efficiency (η_p) can be defined as the fraction of wall absorption among the total loss, (*wall absorption chance)* / (*total loss chance*) = $p_{\mathrm{wall}} \left(1 - R_{\mathrm{wall}}^{\mathrm{p}}\right) \big/ \left(1 - R_{\mathrm{eff}}^{\mathrm{p}}\right)$. The output coupling efficiency (η_{oc}) can also be calculated in a similar way, (*outcoupling chance by the fiber*) / (*total loss chance*) = $p_{\mathrm{oc}} \left(1 - R_{\mathrm{oc}}\right) \big/ \left(1 - R_{\mathrm{eff}}^{\mathrm{em}}\right)$.

Therefore, the efficiency $\eta_p \eta_{oc}$ can be expressed as

$$\begin{aligned} \eta_{\mathrm{p}} \eta_{\mathrm{oc}} &= p_{\mathrm{oc}} p_{\mathrm{wall}} \frac{\left(1 - R_{\mathrm{oc}}\right)\left(1 - R_{\mathrm{wall}}^{\mathrm{p}}\right)}{\left(1 - R_{\mathrm{eff}}^{\mathrm{p}}\right)\left(1 - R_{\mathrm{eff}}^{\mathrm{em}}\right)} \\ &= \frac{p_{\mathrm{oc}} p_{\mathrm{wall}} \left(1 - R_{\mathrm{oc}}\right)\left(1 - R_{\mathrm{wall}}^{\mathrm{p}}\right)}{\left(p_{\mathrm{wall}} R_{\mathrm{wall}}^{\mathrm{p}} + p_{\mathrm{oc}} R_{\mathrm{oc}} + p_{\mathrm{noc}} R_{\mathrm{noc}}\right)\left(p_{\mathrm{wall}} R_{\mathrm{wall}}^{\mathrm{em}} + p_{\mathrm{oc}} R_{\mathrm{oc}} + p_{\mathrm{noc}} R_{\mathrm{noc}}\right)} \end{aligned} . \tag{2}$$

Regarding all the reflectances ($R_{\mathrm{wall}}^{\mathrm{p}}$, $R_{\mathrm{wall}}^{\mathrm{em}}$, R_{oc} , and R_{noc}) and p_{leak} are practically given constants, three variables (p_{wall}, p_{oc} and p_{noc}) are left. Fortunately, we have two more euqtions,

$$p_{\mathrm{wall}} + p_{\mathrm{oc}} + p_{\mathrm{noc}} + p_{\mathrm{leak}} = 1 , \tag{3}$$

and

$$\frac{p_{\text{oc}}}{p_{\text{noc}}} = \frac{m}{1-m} \tag{4}$$

from the Equation (6), where $m = \left(\text{NA} \cdot D_{\text{core}} / D_{\text{a}}\right)^2$ is a given fiber coupling efficiency. Applying Supplementary Equations (3) and (4) to Supplementary Equation (2), the efficiency $\eta_{\text{p}}\eta_{\text{oc}}$ becomes the function of a single variable. Here, we decide to set p_{wall} as a single variable (x) and convert others to the function of it:

$$\begin{aligned} p_{\text{wall}} &= x \\ p_{\text{oc}} &= m(q-x) \\ p_{\text{noc}} &= (1-m)(q-x) \end{aligned} \quad , \tag{5}$$

where $q = 1 - p_{\text{leak}}$ is a constant. Applying Supplementary Equation (5) to Supplementary Equation (2) we get

$$\eta_{\text{p}}\eta_{\text{oc}}(x) = \frac{mx(q-x)(1-R_{\text{oc}})(1-R_{\text{wall}}^{\text{p}})}{\left[xR_{\text{wall}}^{\text{p}} + m(q-x)R_{\text{oc}} + (1-m)(q-x)R_{\text{noc}}\right]\left[xR_{\text{wall}}^{\text{em}} + m(q-x)R_{\text{oc}} + (1-m)(q-x)R_{\text{noc}}\right]} . \tag{6}$$

After simple (but tedious) calculations, the optimized x^* that satisfies $d\left(\eta_{\text{p}}\eta_{\text{oc}}\right)/dx\big|_{x=x^*} = 0$ and maximize the laser efficiency can be found,

$$x^* = \frac{q\gamma_{\text{a}}^2}{\gamma_{\text{a}}^2 + \gamma_{\text{p}}\gamma_{\text{em}}} , \tag{7}$$

and corresponding maximized $\eta_{\text{p}}\eta_{\text{oc}}$ is

$$\max\left(\eta_{\text{p}}\eta_{\text{oc}}\right) = \frac{mq^2}{\gamma_{\text{a}}^2\left(\gamma_{\text{p}} + \gamma_{\text{em}}\right)^2}(1-R_{\text{oc}})(1-R_{\text{wall}}^{\text{p}}) , \tag{8}$$

where $\gamma_{\text{p}} = \sqrt{1-qR_{\text{wall}}^{\text{p}}}$, $\gamma_{\text{em}} = \sqrt{1-qR_{\text{wall}}^{\text{em}}}$, and $\gamma_{\text{a}} = \sqrt{1-qR_{\text{a}}}$, where $R_{\text{a}} = mR_{\text{oc}} + (1-m)R_{\text{noc}}$ is the effective reflectance of the aperture. Applying Supplementary Equation (7) to Equations (4) and (5), the β^* can be deduced as

$$\beta^* = \frac{\gamma_{\text{a}}^2 + (1-q)\gamma_{\text{p}}\gamma_{\text{em}}}{2\left[q\gamma_{\text{p}}\gamma_{\text{em}}\left\{\gamma_{\text{a}}^2 + (1-2q)\gamma_{\text{p}}\gamma_{\text{em}}\right\}\right]^{\frac{1}{2}}} . \tag{9}$$

Notice the Supplementary Equations (9) and (8) become Equations (2) and (3), respectively, when there are no unwanted leaks (q = 1), no loss through the aperture (m = 1), and no back-reflections from the coupling fiber (R_{oc} = 0, or γ_{a} = 1).

The Supplementary Equations (8) and (9) are also validated numerically. Please find the corresponding MATLAB code here: http://doi.org/10.5281/zenodo.4013483

Supplementary Note 2. The effective dwell time and penetration depth

Based on Supplementary Ref. [2], we estimated the effective penetration depth (δD_{cav}) and dwell time ($\delta\tau_{\mathrm{cav}}$) from the absorption and reduced scattering coefficients (μ_{a} and μ_{s}'), taking into account the chance of specular reflection (p_σ),

$$\begin{aligned} \delta D_{\mathrm{cav}} &= \left(1-p_\sigma\right)\frac{1}{\mu_{\mathrm{s}}'} \\ \delta t_{\mathrm{cav}} &= \frac{3}{2}\frac{n_{\mathrm{eff}}}{c}\left(1-p_\sigma\right)\frac{1}{\mu_{\mathrm{s}}'}\left(1+\frac{\mu_{\mathrm{a}}}{\mu_{\mathrm{s}}'}\right)\left(1+\sqrt{\frac{3\mu_{\mathrm{a}}}{\mu_{\mathrm{s}}'}\left(1+\frac{\mu_{\mathrm{a}}}{\mu_{\mathrm{s}}'}\right)}\right)^{-1}, \end{aligned} \tag{10}$$

where $n_{\mathrm{eff}} = \left(n_1 - n_0\right) f_1 + n_0$ is the effective refractive index of the scattering medium, n_1 and n_0 are the refractive indices of gain material and medium (in our cases, it is air), respectively, and f_1 is the volume fraction of gain material. The p_σ is calculated from the solid-angle-averaged Fresnel coefficients of unpolarized light between air-scattering medium interface.

Supplementary References

1. Sato, Y. & Taira, T. Temperature dependencies of stimulated emission cross section for Nd-doped solid-state laser materials. *Opt. Mater. Express* **2**, 1076-1087 (2012).

2. Patterson, M. S., Chance, B. & Wilson, B. C. Time resolved reflectance and transmittance for the noninvasive measurement of tissue optical properties. *Appl. Opt.* **28**, 2331-2336 (1989).
